# Factor analysis of attentional set-shifting performance in young and aged mice

**DOI:** 10.1186/1744-9081-7-33

**Published:** 2011-08-12

**Authors:** Shoji Tanaka, Jared W Young, Jodi E Gresack, Mark A Geyer, Victoria B Risbrough

**Affiliations:** 1 Department of Information & Communication Sciences, Sophia University, Tokyo, Japan; 2 Department of Psychiatry, University of California, San Diego, USA

## Abstract

**Background:**

Executive dysfunction may play a major role in cognitive decline with aging because frontal lobe structures are particularly vulnerable to advancing age. Lesion studies in rats and mice have suggested that intradimensional shifts (IDSs), extradimensional shifts (EDSs), and reversal learning are mediated by the anterior cingulate cortex, the medial prefrontal cortex, and the orbitofrontal cortex, respectively. We hypothesized that the latent structure of cognitive performance would reflect functional localization in the brain and would be altered by aging.

**Methods:**

Young (4 months, n = 16) and aged (23 months, n = 18) C57BL/6N mice performed an attentional set-shifting task (ASST) that evaluates simple discrimination (SD), compound discrimination (CD), IDS, EDS, and reversal learning. The performance data were subjected to an exploratory factor analysis to extract the latent structures of ASST performance in young and aged mice.

**Results:**

The factor analysis extracted two- and three-factor models. In the two-factor model, the factor associated with SD and CD was clearly separated from the factor associated with the rest of the ASST stages in the young mice only. In the three-factor model, the SD and CD loaded on distinct factors. The three-factor model also showed a separation of factors associated with IDS, EDS, and CD reversal. However, the other reversal learning variables, ID reversal and ED reversal, had somewhat inconsistent factor loadings.

**Conclusions:**

The separation of performance factors in aged mice was less clear than in young mice, which suggests that aged mice utilize neuronal networks more broadly for specific cognitive functions. The result that the factors associated with SD and CD were separated in the three-factor model may suggest that the introduction of an irrelevant or distracting dimension results in the use of a new/orthogonal strategy for better discrimination.

## Background

Aging causes declines in cognitive functions. For example, older adults exhibit attentional deficits that can impact their everyday lives [1] and they generally take longer to process information than younger adults [2]. Impairment of memory performance is one of the most noticeable changes in aging; however, not all types of memory decline uniformly. Episodic memory is commonly impaired in the elderly, while implicit and semantic memory remains relatively intact [3]. Executive dysfunction is likely to be a major contributor to the cognitive deficits that are observed with aging because the frontal lobes that mediate executive functions are particularly vulnerable to advancing age [4-7]. Executive control supports adaptation of behavioral responses according to the specific context and requirements of varying situations. The Wisconsin Card Sorting Test (WCST) has been widely used to assess executive function in humans [8]. Subjects are required to adapt behavioral responses to choose the "correct" stimulus array based on sudden rule changes across multiple modalities.

A modification of the WCST, the intradimensional/extradimensional (ID/ED) task, has been used to specifically assess attentional set-shifting abilities [9]. In this task, subjects may initially learn that the color red is the main rule for discriminating between stimuli, and they must form an attentional set using color as the predictive stimulus dimension. The rule is then suddenly changed such that red color no longer predicts the correct array, but instead the color blue. This change is an "intradimensional shift" (IDS). However at another point in the task, the rule is changed such that the stimulus of number is the relevant dimension for discrimination, and color no longer has any predictive value. Thus subjects must rapidly shift their attentional set to a new dimension (i.e. an extra-dimensional shift, EDS), requiring both inhibition of the old rule (color) and acquisition of the new rule (numbers). The EDS is a core component of the WCST, and the main testing procedures of the ID/ED task were taken from the Cambridge Neuropsychological Test Automated Battery (CANTAB) [10,11].

Rat and, more recently, mouse versions of the attentional set-shifting task (ASST) have been developed [12,13]. Like the human versions, the rodent ASST has multiple rule-shifting stages (i.e., IDS and EDS), simple and compound discrimination, and reversal learning, usually using visual, tactile, and odor stimuli as the stimulus modalities. Just as in humans, animals need significantly larger numbers of trials to reach the EDS criterion compared with the IDS criterion, validating the task as a measure of set-shifting [12,13]. Again, similarly to aged humans, aged rats show impairments in EDS performance [14]. A recent ASST study with young and aged mice reported that, while the number of trials needed to reach the criterion did not differ between the young and aged groups, the EDS performance (measured by mean correct latency) was significantly longer and exhibited larger variability in the aged animals [15]. Thus, aged animals appeared to sacrifice the speed of performance in order to maintain the accuracy of performance.

Attentional set-shifting ability is impaired in patients with localized excisions of the frontal lobes, normal elderly controls [9], and patients with schizophrenia [16]. Patients with localized excisions of the frontal lobes have been shown to be selectively impaired in EDS but not IDS. In contrast, both temporal lobe and amygdalo-hippocampectomy patients were unimpaired in either EDS or IDS [9]. Other lesion studies in humans and non-human primates have confirmed that EDS requires an intact dorsolateral prefrontal cortex (DLPFC) [17,18]. Lesion analyses using rats and mice have suggested that IDS, EDS, and reversal learning are mediated by the anterior cingulate cortex; the medial prefrontal cortex (mPFC), the homolog of the primate DLPFC; and the orbitofrontal cortex (OFC), respectively [12,19-23]. These studies demonstrated consistency between humans and rodents in terms of the neural circuits required for attentional set shifting.

We investigated whether the data structure of ASST performance would reflect these functional specificities, and if these distinctions would be altered in aged animals. Here, we report the results of an exploratory factor analysis performed on a dataset taken from a study by Young *et al*. [15]. Because the ASST assesses different cognitive domains (discrimination learning, reversal learning, and set shifting) that are mediated by distinct brain regions, we hypothesized that performances in these domains would be dependent on distinct factors and that aging would modify the latent structure of ASST performance. The preliminary results of this study have been presented in abstract form [24].

## Methods

### Subjects and apparatus

**Figure 1:**
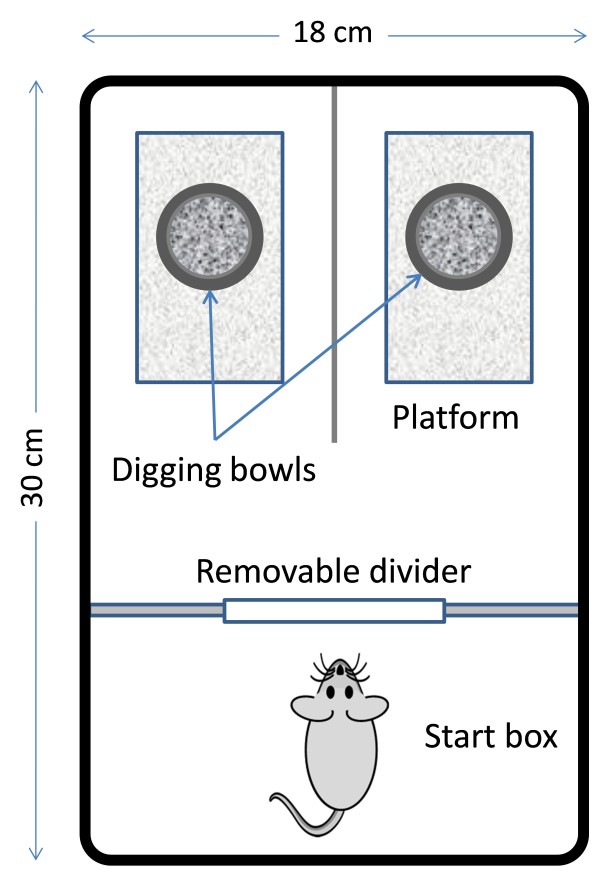
**The test apparatus used in the mouse ASST study (Young et al. 2010)**.

This study analyzed the data from a previously reported study [15]. The animals were either young (5 months, n = 16) or aged (24 months, n = 18) male C57BL/6N mice from the NIA aged rodent colony (Charles River Laboratories, San Diego, California, USA). The experiment started with 27 young and 23 aged mice. While 11 young and 5 aged mice were excluded due to time constraints beyond experimental control, 16 young and 18 aged mice completed all the stages of the ASST and were subjected to the analyses. The test apparatus was an adapted perspex cage (30 × 18 × 12 cm) (Figure 1). Two digging bowls separated by a clear plastic panel were placed in each quarter section. The bowls were placed on platforms (11 × 5 cm) that, in conjunction with odors, were used as cues to guide the selections by the mice.

### Task

The ASST is comprised of 7 stages: simple discrimination (SD); compound discrimination (CD); CD reversal (CDR); IDS; ID reversal (IDR); EDS; and ED reversal (EDR) (Table 1). The SD stage required the animals to distinguish the target stimulus from the irrelevant stimulus, both being presented within a single dimension (e.g., odor). A second, irrelevant dimension (e.g., platform) was introduced in the CD stage and the mice were still required to discriminate between the two stimuli used in the SD task. In the CDR stage, the saliencies of the original stimuli were reversed with the relevant stimulus within the same dimension. The IDS stage required the mice to discriminate the target stimulus from the irrelevant stimulus in the unchanged relevant dimension; however, novel stimulus sets were introduced for both the relevant and the irrelevant dimensions. The IDR stage reversed the saliencies of the IDS stimuli. The EDS stage changed the relevant dimension (e.g., from odor to platform). In the EDR task, the target and irrelevant stimuli were again reversed. Performance was measured both by the number of trials needed to reach the criterion and by the mean correct latency.

**Table 1:** Descriptions of stages within the Attentional Set-Shifting Task

Stage	Description	Dimensions		Exemplar combinations	
		
		Relevant	Irrelevant	Correct	Incorrect
**Simple Discrimination (SD)**	Two stimuli are presented within one dimension (e.g., odor): one stimulus is the target and the other is irrelevant.	**Odor**	--	**O1**	O2

**Compound Discrimination (CD)**	A second dimension (e.g., platform) is introduced but is irrelevant because the subject is still required to discriminate between the two original stimuli.			**O1**/P1	O2/P1
		**Odor**	Platform		
				**O1**/P2	O2/P2

**Compound Discrimination Reversal (CDR)**	The saliencies of the original stimuli are reversed: the target stimulus is now irrelevant, while the irrelevant stimulus becomes the target.			**O2**/P1	O1/P1
		**Odor**	Platform		
				**O2**/P2	O1/P2

**Intradimensional Shift (IDS)**	Novel stimuli are introduced for both dimensions. The target dimension (e.g., odor) remains constant.			**O3**/P3	O4/P3
		**Odor**	Platform		
				**O3**/P4	O4/P4

**Intradimensional Reversal (IDR)**	The saliency of the novel stimuli is reversed: the target stimulus is now irrelevant, while the irrelevant stimulus becomes the target.			**O4**/P3	O3/P3
		**Odor**	Platform		
				**O4**/P4	O3/P4

**Extradimensional Shift (EDS)**	Novel stimuli are introduced for both dimensions, and the target dimension is now changed (e.g., from odor to platform).			**P5**/O5	P6/O5
		**Platform**	Odor		
				**P5**/O6	P6/O6

**Extradimensional Reversal (EDR)**	The saliency of the novel stimuli in the new target dimension is reversed: the target stimulus is now irrelevant while the irrelevant becomes the target.			**P6**/O5	P5/O5
		**Platform**	Odor		
				**P6**/O6	P5/O6

One half of the mice received odor as the initial relevant dimension, while the other half received platform. Odors were ground ginger, nutmeg, garlic, coriander, thyme, and cinnamon. Platforms were sandpaper, wood, neoprene, metal wire, tile, and a scrubber.

### Analysis

We subjected the data to three statistical tests: Student's t-test; correlation analysis; and exploratory factor analysis. These analyses used only the performance scores measured by the number of trials needed to reach the criterion. The exploratory factor analysis used the observed scores of all seven stages of the ASST, assuming that two or three factors would be extracted. A four-factor model was not successful. To obtain factor loadings, we used a promax rotation, a method of oblique rotation that allows factors to be correlated with each other. The promax rotation was preferable to other rotation methods because the assumption of interfactor correlations was reasonable and, more importantly, oblique rotation could reduce cross-loadings [25]. The analyses were performed using R (The R Project for Statistical Computing), a software environment for statistical computing and graphics.

## Results

### Correlations

**Figure 2:**
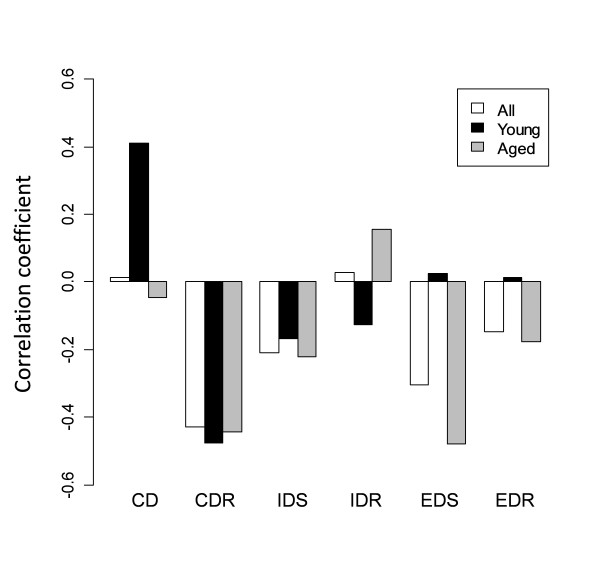
**Pearson's correlations of SD with other ASST variables**.

The correlation matrices of ASST performances in young and aged mice are listed in Table 2. The correlations of SD with other ASST variables are shown in Figure 2. Both the young and the aged groups showed significant negative correlations between SD and CDR. The young group, but not the aged group, showed a positive correlation between SD and CD. The aged group, but not the young group, showed a negative correlation between SD and EDS.

**Table 2:** Pearson's correlations for ASST performance

*Young*	SD	CD	CDR	IDS	IDR	EDS	EDR
**SD**	1	0.41	-0.48	-0.17	-0.13	0.03	0.01
**CD**	0.41	1	-0.19	0.17	-0.26	-0.13	-0.08
**CDR**	-0.48	-0.19	1	0.15	-0.05	0.23	0.08
**IDS**	-0.17	0.17	0.15	1	-0.10	0.07	0.48
**IDR**	-0.13	-0.26	-0.05	-0.10	1	0	-0.30
**EDS**	0.03	-0.13	0.23	0.07	0	1	0.19
**EDR**	0.01	-0.08	0.08	0.48	-0.30	0.19	1

***Aged***	**SD**	**CD**	**CDR**	**IDS**	**IDR**	**EDS**	**EDR**

**SD**	1	-0.05	-0.45	-0.22	0.16	-0.48	-0.18
**CD**	-0.05	1	-0.37	0.56	0.59	-0.23	-0.02
**CDR**	-0.45	-0.37	1	-0.04	-0.06	0.34	0.22
**IDS**	-0.22	0.56	-0.04	1	0.52	-0.13	0.52
**IDR**	0.16	0.59	-0.06	0.52	1	-0.37	0.18
**EDS**	-0.48	-0.23	0.34	-0.13	-0.37	1	0.04
**EDR**	-0.18	-0.02	0.22	0.52	0.18	0.04	1

### Two-factor model

**Figure 3:**
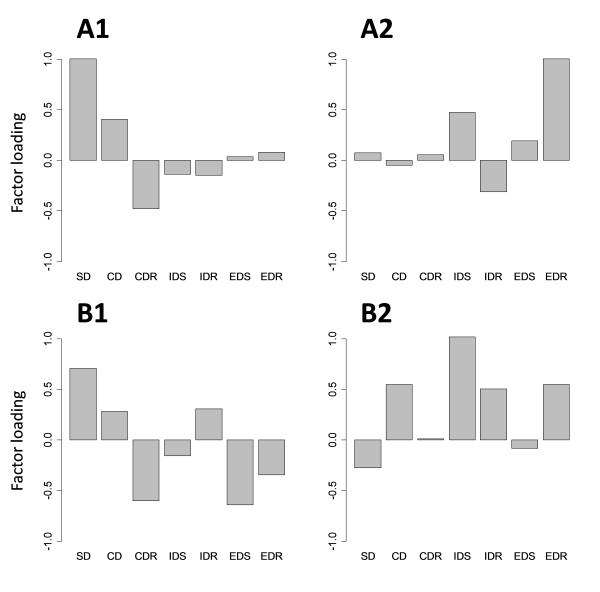
**Factor loading profiles across the ASST stages for the two-factor models with promax rotation**. A1 and A2: Two factors in young mice. The cumulative variance is 0.41 and p = 0.90. B1 and B2: Two factors in aged mice. The cumulative variance is 0.51 and p = 0.38.

The extracted two-factor model is shown in Table 3 and Figure 3. In young mice, SD, CD, and CDR had high factor loadings on the first factor, which is termed the "discrimination factor." The remaining stages (IDS, IDR, EDS, and EDR) had high factor loadings on the second factor. The aged mice did not show such a clear separation, but the IDS had the highest loading on the second factor. Because the IDS was the first shift in the ASST, the second factor is termed the "shifting factor."

**Table 3:** Factor loadings of the ASST stages for the two-factor models with promax rotation

*Young*	Factor1	Factor2	*Aged*	Factor1	Factor2
**SD**	1.006		**SD**	0.708	-0.277
**CD**	0.409		**CD**	0.283	0.550
**CDR**	-0.475		**CDR**	-0.602	0
**IDS**		0.475	**IDS**	-0.156	1.021
**IDR**		-0.310	**IDR**	0.308	0.506
**EDS**		0.194	**EDS**	-0.640	0
**EDR**		1.006	**EDR**	-0.343	0.551

Cutoff = 0.15.

### Three-factor model

**Figure 4:**
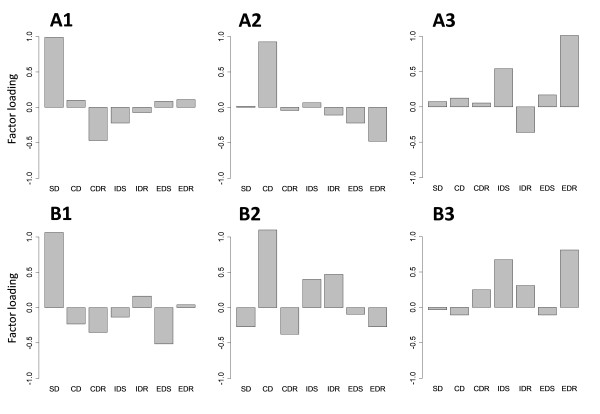
**Factor loading profiles across the ASST stages for the three-factor models with promax rotation**. A1, A2 and A3: Three factors in young mice. The cumulative variance is 0.56 and p = 0.78. B1, B2 and B3: Three factors in aged mice. The cumulative variance is 0.69 and p = 0.55.

The three-factor model is shown in Table 4 and Figure 4. In this model, SD and CD loaded on distinct factors in both young and aged mice. Because the difference between SD and CD tasks was the absence or presence of distractors, discrimination in a distracting environment can be functionally differentiated from discrimination without distractors. Therefore, the first two factors of this three-factor model are termed the "discrimination factor" and the "undistracted-discrimination factor." The third factor was similar to the second factor in the two-factor model in that both IDS and EDR had high factor loadings in both young and aged mice. EDS did not have consistent loadings between the groups. We term the third factor the "shifting factor" because IDS was the first shifting stage in the ASST. As with the two-factor model, the factor loading profiles were less clear in aged mice than in young mice.

**Table 4:** Factor loadings of the ASST stages for the three-factor models with promax rotation

*Young*	Factor1	Factor2	Factor3	*Aged*	Factor1	Factor2	Factor3
**SD**	0.991			**SD**	1.066	-0.271	
**CD**		0.926		**CD**	-0.229	1.102	
**CDR**	-0.469			**CDR**	-0.351	-0.376	0.251
**IDS**	-0.221		0.539	**IDS**		0.405	0.675
**IDR**			-0.361	**IDR**	0.165	0.474	0.310
**EDS**		-0.219	0.169	**EDS**	-0.511		
**EDR**		-0.476	1.012	**EDR**		-0.270	0.811

Cutoff = 0.15.

### Distractibility

Previous studies have shown that the presence of distractors increases error rates during cognitive tasks in aged subjects [26,27]. We estimated the aging effects on distractibility by subtracting SD from CD, to approximate the increased number of trials needed to learn the discrimination when distracters are present. The aged group had a higher mean value of "CD minus SD" (mean: young = -5.56, aged = 0.28), with a trend toward a significant effect of age (p = 0.065, one-tailed t-test).

## Discussion

The two-factor model clearly showed a separation of performance during discrimination stages (SD, CD, and CDR) from the remaining stages (IDS, IDR, EDS, and EDR) in young mice only. CDR had a negative factor loading, whereas SD had a positive factor loading in both groups. The correlation analysis also confirmed negative correlations between SD and CDR (Figure 2). This negative relationship is interesting because the CDR involves discrimination but is also the first reversal learning stage in the ASST. The negative correlation between SD and CDR indicates that mice with high SD performance performed poorly with reversal learning. There might be a trade-off relationship between simple discrimination and reversal learning. Therefore, the "discrimination factor," on which the SD, CD, and CDR load, might also include a tendency for "perseveration."

The reciprocal SD-CDR relationship observed in our mouse study is consistent with a similar study using ASST with young and aged rats [14]. Those researchers used 4- to 5-month-old and 27- to 28-month-old male Long-Evans rats (n = 26) in a similar ASST study, but no CDR or EDR stage was employed. In the rat study, IDR was the first reversal task, while CDR was the first in the mouse study that we analyzed. There was a negative correlation between SD and IDR in the rat study, a finding that is similar to our study. Their results differ from ours in that the correlation was negative only in the young rats (young = -0.728; aged = -0.155). In contrast, we found a negative correlation between SD and CDR in both groups (young = -0.477; aged = -0.455), which suggests that mice with better SD performed worse in CDR, irrespective of age.

The three-factor model further separated CD from SD, which suggests that the addition of irrelevant stimuli required a new strategy for discrimination. This separation was shown in both groups of mice. Notably, the factor loadings showed that the separation of CD from SD was enhanced in aged mice when compared with young mice (Table 4). This clearer separation may be due to increased distractibility in aged mice. The degree of distractibility was estimated by calculating the difference between SD and CD. Higher values of "CD minus SD" appeared to be indicative of higher distractibility. The aged group had a higher mean value of "CD minus SD," with a trend toward significance between the two groups. This finding indicates that, unlike young mice, aged mice were affected by the addition of an irrelevant stimulus. These data are consistent with human studies, indicating that aged mice have higher distractibility or lower robustness of executive functioning when compared with young mice [28-30].

Because IDS, EDS, and reversal learning may be mediated by distinct brain regions [12,19-21,23], we expected that these variables would load on distinct factors. IDS, EDS, and CDR did indeed load on distinct factors in both groups. The separation of these factors is meaningful because CDR was the first reversal learning stage in the ASST. However, the factor loadings of EDS were fairly low for all factors extracted. It is unclear why CDR, IDR, and EDR did not load on a single factor; therefore, the extracted three-factor model had unexpected factor loadings. There are several possible reasons for these results. First, the mice may not have formed an attentional set; however, this explanation is unlikely because both the young and the aged mice showed significant increases in the number of trials needed to reach the criterion for the EDS stage when compared with the IDS stage, regardless of the initial perceptual dimension [15]. This finding suggests the successful formation of an attentional set in this group. Second, despite the hypothesized functional localization in the brain, these regions likely interact to perform the ASST stages. It has been suggested that cognitive processing is mediated by a network [31-34]. The network involved in ASST performance would include the mPFC, OFC, and other regions, and interaction among the involved regions in the network might have blurred the separation of factors. The capability of factor analysis to extract a functional structure in such a distributed network is an interesting issue that needs to be addressed. Third, a small sample size (n = 16 for young mice and n = 18 for aged mice) was used in this analysis. The same analysis with a larger sample size should be performed to determine whether the sample size was a limiting factor. It would be interesting to see whether a factor analysis with a larger sample size would result in a better model with more consistent factor loadings. Finally, there is evidence in rats and mice that reversal learning improves over repeated exposure, with fewer trials required at EDR vs. CDR [15,23,35]. This 'learning to reverse' might incorporate other neuroanatomical structures that are not specific to reversal learning, hence the lack of commonality in reversal loading.

In our study, the overall deterioration in the cognitive function of aged mice was not marked. However, while the number of trials needed to reach the criterion was not different between groups for EDS, aged mice exhibited significantly longer mean correct latencies during this task (p < 0.05) [15]. This result indicates that the aged mice have reduced processing speed only during the EDS stage. Thus, when compared with young mice, aged mice might require more time to perform the EDS (i.e. speed-accuracy trade off) [15]. Magnetic resonance imaging studies in humans performing an executive function task reported a broader activation of brain regions in older subjects when compared with younger subjects who were performing at comparable levels. This result suggests that alternate networks are being recruited in older subjects [36-39] and that the elderly use a different strategy when performing a cognitive task [39-42]. Potential neurochemical differences that might account for alterations in aging performance in ASST are numerous. In young animals, dopamine signaling is critical for EDS performance. For example, amphetamine-sensitized rats or rats that acquired amphetamine self-administration exhibited impaired EDS but not IDS performance [43-45]. Treatment with tolcapone, a catechol-O-methyltransferase inhibitor that increases dopamine and norepinephrine levels in the frontal cortex, improved EDS performance in rats [46]. This latter study suggests that catecholamine transmission in the rat mPFC is critically involved in set-shifting functions. A recent study in rats has suggested that remodeling of mesocortical dopaminergic fibers is involved in age-associated cognitive decline [47]. Other studies have also demonstrated a specific involvement of noradrenergic transmission in ASST performance [48-51], while serotonin modulation may affect reversal learning components in this task but not EDS performance [52]. Interestingly, administration of naltrexone (2 mg/kg, i.p.), an opioid antagonist, has been shown to reverse aging-related deficits in EDS [53], suggesting that altered endorphin signaling plays a role in aging-induced decline in set shifting. Research on the complex underpinnings of age-related deficits in attentional set shifting is in its infancy; however, these data suggest that there are a number of potential systems that could influence aging effects on this task.

## Conclusion

The exploratory factor analysis applied to the mouse ASST data extracted two- and three-factor models. The two-factor model clearly separated discrimination performance (SD and CD) from the remaining ASST stages in young mice. This tendency was obscured in the aged mice, suggesting that they utilize a less selective network in the brain for cognitive functions. The three-factor model further separated CD from SD. The result that the factors associated with SD and CD were separated in the three-factor model may suggest that the introduction of an irrelevant or distracting dimension results in the use of a new/orthogonal strategy for better discrimination. This model, however, did not show consistent separation of the factors associated with IDS, EDS, and reversal learning in either young or aged mice, contrary to the hypothesis that these functions are mediated by distinct brain regions. Whether the inconsistent separation of factors was due to possible interaction among the brain regions or to the small sample size needs to be clarified by a future analysis using a larger sample size.

## Competing interests

The authors declare that they have no competing interests.

## Authors' contributions

ST carried out the whole analysis. JWY, JEG, MAG, and VBR carried out the original animal experiment. All authors read and approved the final manuscript.
